# *Cedrus atlantica* extract exerts antiproliferative effect on colorectal cancer through the induction of cell cycle arrest and apoptosis

**DOI:** 10.1002/fsn3.2786

**Published:** 2022-02-21

**Authors:** Chih‐Yuan Huang, Ju‐Huei Chien, Kai‐Fu Chang, Chih‐Yen Hsiao, Ya‐Chih Huang, Yi‐Ting Chen, Ming‐Yi Hsu, Ming‐Chang Hsieh, Nu‐Man Tsai

**Affiliations:** ^1^ 71664 Devision of Nephrology Department of Internal Medicine Ditmanson Medical Foundation Chia‐Yi Christian Hospital Chia‐Yi Taiwan, ROC; ^2^ 71664 Department of Sport Management College of Recreation and Health Management Chia Nan University of Pharmacy and Science Tainan Taiwan, ROC; ^3^ Department of Research Taichung Tzu‐Chi Hospital Buddhist Tzu‐Chi Medical Foundation Taichung Taiwan, ROC; ^4^ 63340 Department of Medical Laboratory Science and Biotechnology Central Taiwan University of Science and Technology Taichung Taiwan, ROC; ^5^ 63276 Department of Medical Laboratory and Biotechnology Chung Shan Medical University Taichung Taiwan, ROC; ^6^ 71664 Department of Hospital and Health Care Administration Chia Nan University of Pharmacy and Science Tainan Taiwan, ROC; ^7^ 63276 Institute of Medicine Chung Shan Medical University Taichung Taiwan, ROC; ^8^ 63276 Department of Nursing Chung Shan Medical University Taichung Taiwan, ROC; ^9^ 63276 Department of Nursing Chung Shan Medical University Hospital Taichung Taiwan, ROC; ^10^ 63276 Clinical Laboratory Chung Shan Medical University Hospital Taichung Taiwan, ROC; ^11^ Department of Life‐and‐Death Studies Nanhua University Chiayi Taiwan, ROC

**Keywords:** antiproliferation, apoptosis, *Cedrus atlantica*, colorectal cancer, synergistic inhibition

## Abstract

*Cedrus atlantica* is a tree species found in Morocco with many clinical benefits in genitourinary, musculoskeletal, and skin systems. Previous studies have reported that extracts of *Cedrus atlantica* have antioxidant, antimicrobial, and anticancer properties. However, its role in colorectal cancer (CRC) remains unclear. The present study investigated the effects and underlying mechanisms of *Cedrus atlantica* extract (CAt) using HT‐29 (human colorectal adenocarcinoma) and CT‐26 CRC cell lines. The 3‐(4,5‐dimethylthiazol‐2‐yl)‐2,5‐diphenyltetrazolium bromide (MTT) assay was performed to measure cell viability. Flow cytometry analysis and terminal deoxynucleotidyl transferase dUTP nick‐end labeling (TUNEL) assay were used to study the cell cycle and cell apoptosis, respectively. The expression of cell cycle and apoptosis‐associated proteins was detected by western blotting or immunohistochemical (IHC) staining. CAt showed significant inhibitory effects on the proliferation of HT‐29 and CT‐26 cells, and combined with the clinical drug, 5‐fluorouracil (5‐FU), exhibited synergistic effects. CAt induced cell cycle arrest at the G0/G1 phase through the upregulation of p53/p21 and the downregulation of cyclin‐dependent kinases (CDKs)/cyclins. In addition, CAt‐treated cells exhibited chromatin condensation, DNA fragmentation, and apoptotic bodies, which are typical characteristics of apoptosis activated via both the extrinsic (Fas ligand (FasL)/Fas/caspase‐8) and intrinsic (Bax/caspase‐9) pathways. Importantly, CAt suppressed tumor progression and prolonged the life span of mice within a well‐tolerated dose. Therefore, our findings provide novel insights into the use of CAt for the treatment of CRC.

## Graphical abstract

*Cedrus atlantica* extract (CAt) exerts antiproliferative effect on colorectal cancer (CRC) through the induction of cell cycle arrest and apoptosis, suggesting that CAt may be exploited for the development of novel anticancer agents or dietary supplements against CRC.

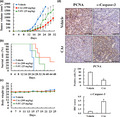

## 1 INTRODUCTION

Colorectal cancer (CRC) is one of the most common cancers worldwide: global cancer statistics show that the prevalence of CRC ranks third in the world in 2018, with approximately 1.8 million people diagnosed with CRC each year (Bray et al., 2018). It is estimated that by 2030, 2.2 million new cases of CRC will be diagnosed and 1.1 million will die from CRC (Arnold et al., 2017). Most CRC cases are sporadic, and 18%–35% of cases are from family inheritance, indicating that the environment and genetic background are relevant to the occurrence of CRC (Lynch & de la Chapelle, 2003; Rawla et al., 2019). Among the environmental risk factors associated with CRC, the most important are high‐calorie diets of rich animal fat, smoking, increased alcohol consumption, and insufficient intake of vegetables, fruits, and fibers. Despite advances in CRC screening, approximately 35% of colorectal cancer patients present with stage IV metastasis at the time of diagnosis, while 20%–50% of patients with stage II or III metastasis will develop into stage IV as the disease progresses (Zacharakis et al., 2010). Although CRC therapy has improved, the 5‐year survival rate of patients with distant metastasis is still only 10%–15%.

Methods commonly used in the treatment of CRC include surgery, radiotherapy, chemotherapy, targeted therapy, and immunotherapy, which are based on tumor size, location, cancer stage, and patient health status. 5‐Fluorouracil (5‐FU), irinotecan (Camptosar), oxaliplatin (Eloxatin), capecitabine (Xeloda), and trifluridine/tipiracil (Lonsurf) are commonly used to treat CRC, and they can be used alone or in combination to increase response rates and reduce the development of drug resistance (Kim, 2015). However, the long‐term use of these drugs can cause serious side effects and reduce the quality of life. Recent studies have indicated that many natural products are targeted agents that can induce tumor cell apoptosis, inhibit proliferation, initiate cell cycle arrest, and have great anticancer potential (Li et al., 2016).

For centuries, extensive research has been conducted on drug discovery and development from plant extracts and natural products, which contain numerous molecules with proven cytotoxicity inducing apoptosis via different signaling pathways against cancers (Benarba & Pandiella, 2018). For example, anticancer compounds such as Vinca alkaloids isolated from *Catharanthus roseus* (Shams et al., 2009), podophyllotoxin isolated from *Podophyllum peltatum* (Ardalani et al., 2017), camptothecin isolated from *Camptotheca acuminata* (Ran et al., 2017), taxol isolated from *Taxus brevifolia* (Kuriakose et al., 2020), and their derivatives are widely used as first‐line and second‐line cancer therapies.

*Cedrus* species (Pinaceae) classified by their morphological diversities include *C. atlantica* in Morocco and Algeria, *C. libani* in Lebanon, Syria, and Turkey, *C. brevifolia* in Cyprus, and *C. deodara* in the Himalaya Mountains (Panetsos et al., 1992). Essential oils extracted from different species of *Cedus* have traditionally been used in aromatherapy for many clinical benefits of the genitourinary, musculoskeletal, and skin systems (Lovell, 1998; Gabriel Mojay, 2002; G. Mojay, 2004). *Cedrus atlantica* is the largest remaining population and the main forest species in Morocco used for timber production, and sawdust is usually refined by hydrodistillation to provide essential oils. It exerts antimicrobial (Dakir et al., 2005; Shin, 2003) and anticancer (Chang et al., 2021; Huang et al., 2020; Saab et al., 2012) activities and alleviates pain behavior via inhalation (Martins et al., 2015). However, there is currently a lack of information regarding the potential anticancer properties of *C. atlantica* extract (CAt) against CRC. The present study assessed the anticancer effects of CAt in CRC cells and investigated the underlying molecular mechanisms in vitro and in vivo.

## 2 MATERIALS AND METHODS

### 2.1 Antibodies, chemicals, and regents

Antibodies used to detect p53, p‐p53, Rb, p‐Rb, p21, proliferating cell nuclear antigen (PCNA), CDK4, cyclin D1, cyclin‐dependent kinase 2 (CDK2), cyclin A, cyclin B1, Fas, FasL, caspase‐8, Bax, caspase‐9, and caspase‐3 were purchased from Santa Cruz Biotechnology (CA, USA), and β‐actin was purchased from iReal Biotechnology (Hsinchu, Taiwan).

The preparation of CAt was commissioned to Phoenix (New Jersey, USA) according to the following conditions: the bark of *Cedrus atlantica* was extracted through steam distillation at a flow rate of approximately 7.2 ml/min at 100___105°C for 90 min (Chang et al., 2021). CAt and 5‐FU (Sigma‐Aldrich, St. Louis, MO, USA) were dissolved in dimethyl sulfoxide (DMSO) and diluted in fresh medium. The final concentration of DMSO for cell treatment was <1%.

### 2.2 Cell lines and culture

HT‐29 (human colorectal adenocarcinoma) cells were purchased from the American Type Culture Collection (ATCC, Rockville, MD, USA). CT‐26 (mouse colorectal carcinoma), SVEC (mouse vascular endothelial), and MDCK (canine kidney epithelial) were obtained from the Bioresource Collection and Research Center (BCRC, Hsinchu, Taiwan). The cells were maintained in Dulbecco's modified Eagle's medium (DMEM) (HT‐29, SVEC, and MDCK) or RPMI‐1640 (Roswell Park Memorial Institute) (CT‐26) medium supplemented with 10% fetal bovine serum (FBS) (Gibco BRL, Gaithersburg, MD, USA), penicillin/streptomycin solution (Gibco), HEPES (4‐(2‐hydroxyethyl)‐1‐piperazineethanesulfonic acid) (Gibco), and pyruvate (Gibco) at 37°C, and incubated in a humidified 5% CO_2_ atmosphere. The status of TP53 exon8 in HT‐29 cells was mutant type (R273H) using automated nucleic acid extraction (AccuBioMed Co., Ltd., Taipei, Taiwan) and sequencing using Femtopath Human TP53 Primer Sets (HongJing Biotech, Taipei, Taiwan).

### 2.3 Cell viability assay

Cell viability was determined using a modified 3‐(4,5‐dimethylthiazol‐2‐yl)‐2,5‐diphenyltetrazolium bromide (MTT) assay. Briefly, 5,000 cells were seeded into 96‐well plates, treated with CAt (0–100 μg/ml) or 5‐FU (0–50 μg/ml), and incubated at 37°C for 24, 48, and 72 h. Then, 100 μl of MTT solution in medium (400 μg/ml) (Sigma‐Aldrich) was added to each well and the cells were incubated for 4 h. A microplate reader (Spec384; Molecular Devices) was used to measure the absorbance at 550 nm. Cell viability was calculated as the OD percentage relative to the control (100%).

### 2.4 Determination of the drug combination effect

HT‐29 cells were seeded in 96‐well culture plates (5 × 10^3^ cells/well) for 24 h, and treated with CAt (0, 10, 20, 40, and 80 μg/ml) combined with 1.5 μg/ml 5‐FU or 5‐FU (0, 1, 2, 4, and 8 μg/ml) combined with 30 μg/ml CAt for 48 h. Cell viability was determined using an MTT assay. The drug interactions were determined based on the combination index (CI) to evaluate the occurrence of synergism (CI < 1), an additive effect (CI = 1), and antagonism (CI > 1), using CompuSyn (ComboSyn, Inc., Paramus, NJ, USA).

### 2.5 Analysis of cell cycle distribution

HT‐29 cells were plated in a 100‐mm culture dish at a density of 2 × 10^6^ cells and cultured for 24 h. They were then incubated with 35 μg/ml CAt for 0, 6, 12, 24, and 48 h; CAt (25, 35, and 45 μg/ml) for 24 h. Cells were collected and stained with propidium iodide (PI, 40 μg/ml; Sigma‐Aldrich) supplemented with ribonuclease A (RNase A) (100 μg/ml; Sigma‐Aldrich), at 4°C overnight in the dark. The proportions of cells in different phases (G0/G1 phase, S phase, and G2/M phase) and the percentage of cells in the subG1 phase were evaluated using FACScan (Beckton Dickinson, USA) and Kaluza Flow Cytometry Analysis Software (Software Version 1.2, Beckman Coulter, USA).

### 2.6 Detection of the cell apoptosis

Apoptosis was determined by the terminal deoxynucleotidyl transferase dUTP nick‐end labeling (TUNEL) assay using the Situ Cell Death Detection Kit (Roche, Mannheim, Germany). Cells were treated with 35 μg/ml CAt for 48 h, collected, and washed with phosphate‐buffered saline (PBS). After fixation with 10% formaldehyde, the cells were smeared and dried on silane‐coated glass slides. Then, cells or deparaffinized sections were rehydrated with PBS, inactivated endogenous peroxidase using 3% H_2_O_2_ in methanol, and permeabilized using 0.1% Triton X‐100 in 0.1% sodium citrate on ice. Samples were incubated with TUNEL solution for 2 h at 37°C, counterstained with PI, and observed under a fluorescence microscope (Axioskop 2; Zeiss) at 400× magnification.

### 2.7 Western blot analysis

HT‐29 cells were seeded at a density of 2 × 10^6^ cells in a 100‐mm dish. The next day, the cells were treated with 35 μg/ml CAt for 0, 6, 12, 24, and 48 h. Cell lysates were prepared by adding radioimmunoprecipitation (RIPA) buffer containing a protease inhibitor (Bio Basic Inc., Canada) and phosphatase inhibitor (Bionovas, Toronto, Canada), and incubated on ice for 30 min. The extracted proteins were estimated according to the protocol of bicinchoninic acid protein assay kit (Pierce, Rockford, IL, USA). Approximately 20 μg of the proteins was separated by 8%–12.5% sodium dodecyl sulfate–polyacrylamide gel electrophoresis (SDS–PAGE) and transferred to 0.22‐μm polyvinylidene difluoride (PVDF) membranes (Pall Corporation, USA). The PVDF membranes were blocked with 5% nonfat dry milk for 30 min, followed by probing the membranes with blocking buffer‐diluted specific primary antibodies (1:1,000 dilution) at 4°C overnight with continuous shaking. The membranes were washed three times with 0.5% Tween‐20 in Tris‐buffered saline, incubated with biotin‐conjugated secondary antibodies (Santa Cruz, CA, USA) for 2 h, followed by interaction with peroxidase‐conjugated streptavidin (Jackson ImmunoResearch Inc., USA) for 1 h. After washing and treatment with an enhanced chemiluminescence reagent (ECL) (T‐Pro Biotechnology, Taiwan), the blots were scanned and analyzed using a chemiluminescence imaging analyzer (GE LAS‐4000; GE Healthcare Life. Sciences, NJ, USA) and ImageJ software 1.47t (National Institutes of Health, Bethesda, MD, USA). The density ratio of sample to control was calculated as follows: density ratio = (normalized sample/normalized control).

### 2.8 Animal study

CT‐26 cells were used to construct an animal model. The animal experiments were performed at the Chung Shan Medical University (CSMU) and following the Guide for the Care and Use of Laboratory Animals and approved by the Institutional Animal Care and Use Committee (IACUC) of CSMU (Approval No. CSMU‐IACUC‐1543). BALB/c mice (10–12 weeks, 22–24 g) were purchased from the National Laboratory Animal Center (Taipei, Taiwan). CT‐26 cells (1 × 10^6^) were subcutaneously injected into the right flank of the mice. The vehicle group (*n* = 4) and CAt group (*n* = 6) received solvent (100 μl of mineral oil) and 200 mg/kg of CAt, respectively (every 2 days for 20 times, subcutaneous injection). 5‐FU (*n* = 4) was intraperitoneally injected 3 times a week at the dose of 25 mg/kg for 21 days as positive control (Cho et al., 2020). Tumor size (L × H × W × π/6 mm^3^) and body weight were recorded every 2 days. Mice were sacrificed using carbon dioxide when the tumor volume was greater than 1,500 mm^3^. The tumors and organs were fixed with 4% formaldehyde, embedded in paraffin, and sliced for hematoxylin and eosin (H&E) and immunohistochemical (IHC) staining.

### 2.9 H&E and immunohistochemistry stain

In H&E staining, the deparaffinized sections (7 μm) were stained with hematoxylin and eosin (Muto Pure Chemicals, Tokyo, Japan), after which the tissue morphology was observed and photographed under a bright field microscope. For IHC staining, the sections (4 μm) were heated at 65°C for 30 min, deparaffinized, and hydrated through a series of xylene and alcohol baths. The slides were microwaved in an antigen retrieval solution (Thermo Fisher Scientific Inc., MA, USA) for 5 min. The sections were immersed in 3% H_2_O_2_ for 10 min to inactivate endogenous peroxidase activity and then blocked with 10% bovine serum albumin (BSA) for 30 min. Thereafter, immunohistochemical staining was performed using rabbit anti‐PCNA antibody and mouse anti‐caspase‐3 antibody and incubated at 4°C overnight. After washing with 0.1% Tween‐20 in PBS for 3 times, the antibody‐binding proteins were detected and visualized using Super Sensitive Polymer‐HRP IHC Detection System (BioGenex, CA, USA) and 3,3'‐diaminobenzidine (DAB) substrate (BioGenex, CA, USA). Finally, the sections were photographed using a bright field microscope and scored using the Quickscore method performed according to a previously published protocol (Chang et al., 2021).

### 2.10 Statistical analysis

The results are expressed as the mean ± SD (in vitro) or mean ± SEM (in vivo). Statistical analysis was performed using an unpaired Student's *t*‐test or one‐way analysis of variance (ANOVA) to analyze the differences between each group, and the Kaplan–Meier method was used for the survival rate analysis. Statistical significance was set at *p* < .05. Experiments were repeated at least three times in duplicate or triplicate.

## 3 RESULTS

### 3.1 Effects of CAt on the viability of CRC cells

**FIGURE 1:**
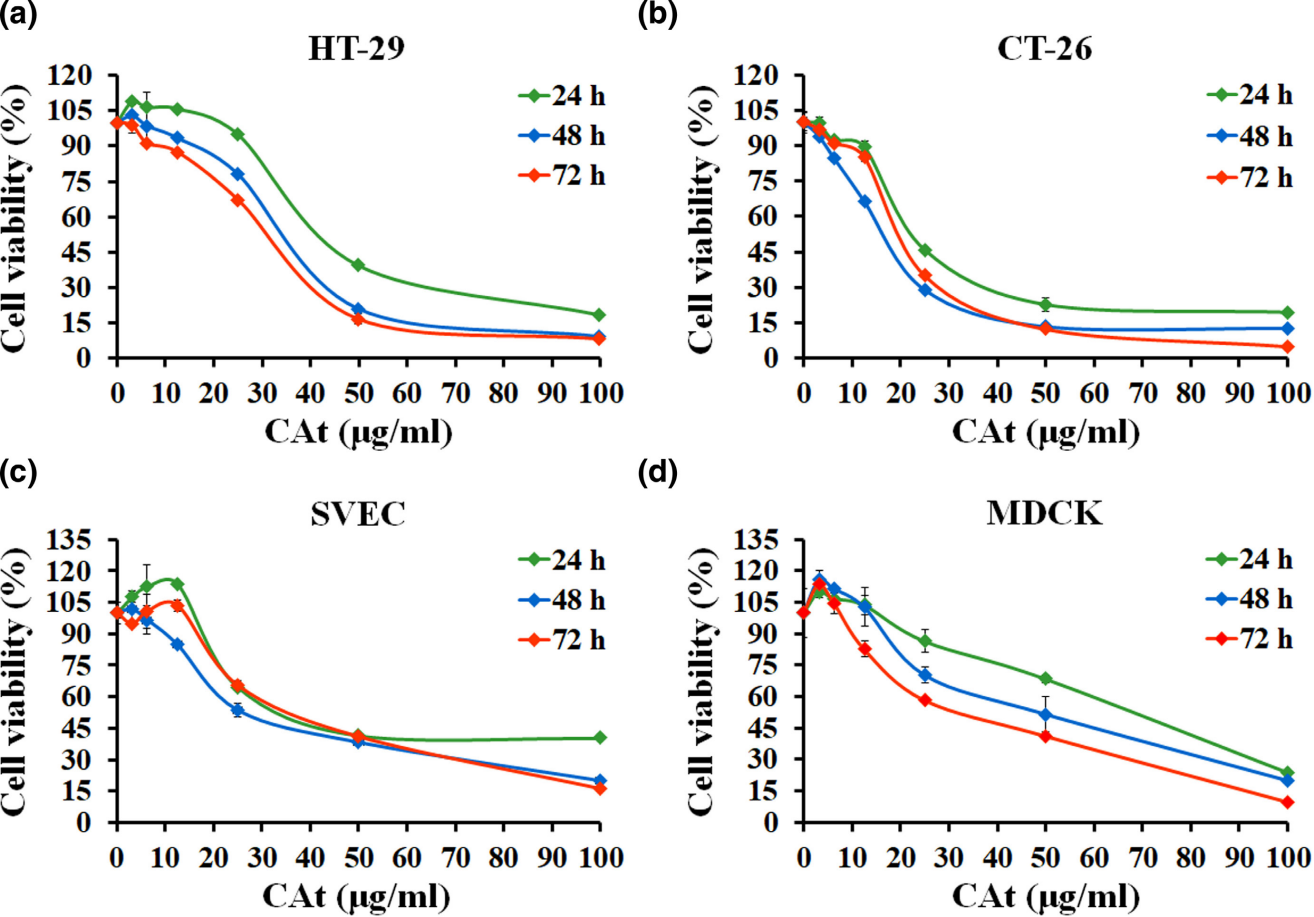
*Cedrus atlantica* extract (Cat) inhibited the cell proliferation of colorectal cancer (CRC) cells. Human colorectal adenocarcinoma (HT‐29) (a), mouse colorectal carcinoma (CT‐26) (b), mouse vascular endothelial (SVCE) (c), and canine kidney epithelial (MDCK) (d) cells were treated with CAt (0, 3.125, 6.25, 12.5, 25. 50, and 100 μg/ml) for 24, 48, and 72 h. The 3‐(4,5‐dimethylthiazol‐2‐yl)‐2,5‐diphenyltetrazolium bromide (MTT) assay was used to monitor cell viability. Data were shown as the mean ± SD of three independent experiments

In our previous study, the major components of CAt included α‐cedrene (37.98%), cedrol (23.03%), thujopsene (19.45%), γ‐muurolene (6.68%), and cuparene (2.14%), identified using a gas chromatography–mass spectrometry (GC–MS) spectrometer (Chang et al., 2021). To evaluate the anticancer activity of CAt against CRC cells, we performed an MTT cell proliferation assay on two CRC cell lines, HT‐29 and CT‐26. First, different concentrations of CAt (0–100 μg/ml) were used to treat CRC and normal cells (SVEC and MDCK). Cell viability was measured at different time points (24, 48, and 72 h), and a cell growth inhibition curve was constructed (Figure 1). The results showed that CAt inhibited the proliferation of CRC cells in a concentration‐ and time‐dependent manner, but had less effect on normal cells. Furthermore, the half‐maximal inhibitory concentration (IC_50_) of all cells (HT‐29, CT‐26, SVEC, and MDCK) was determined at 48 h, and the values were 31.21 ± 1.36 μg/ml, 19.77 ± 0.7 μg/ml, 45.62 ± 0.88 μg/ml, and 69.71 ± 2.1 μg/ml, respectively (Table 1). Comparison with normal cells indicated that CAt had more drug selectivity to CRC cells, but this effect was not observed with 5‐FU. Based on the data, it can be concluded that CAt showed effective inhibitory effects in CRC cells but not in normal cells.

**TABLE 1:** The IC_50_ values of *Cedrus atlantica* extract (Cat) in colorectal carcinoma (CRC) and normal cells

Cell line	Tumor type	CAt	5‐FU
HT‐29	hu colorectal adenocarcinoma	31.21 ± 1.36*^,#^	7.86 ± 1.65
CT‐26	mo colorectal carcinoma	19.77 ± 0.70*^,#^	<3.125
SVEC	mo vascular endothelial cell	45.62 ± 0.88^#^	<3.125
MDCK	canine kidney epithelial cell	69.71 ± 2.10^#^	12.35 ± 0.39

Note

The half‐maximal inhibitory concentration (IC_50_) value was measured from the cell viability assay. Values are mean ± SD (μg/ml) at 48 h for at least three independent experiments. hu, human; mo, mouse. *p < .05 versus normal cells. ^#^ *p* < .05 versus 5‐FU treatment.

### 3.2 CAt combined with 5‐FU revealed synergistic effects in CRC cells

**FIGURE 2:**
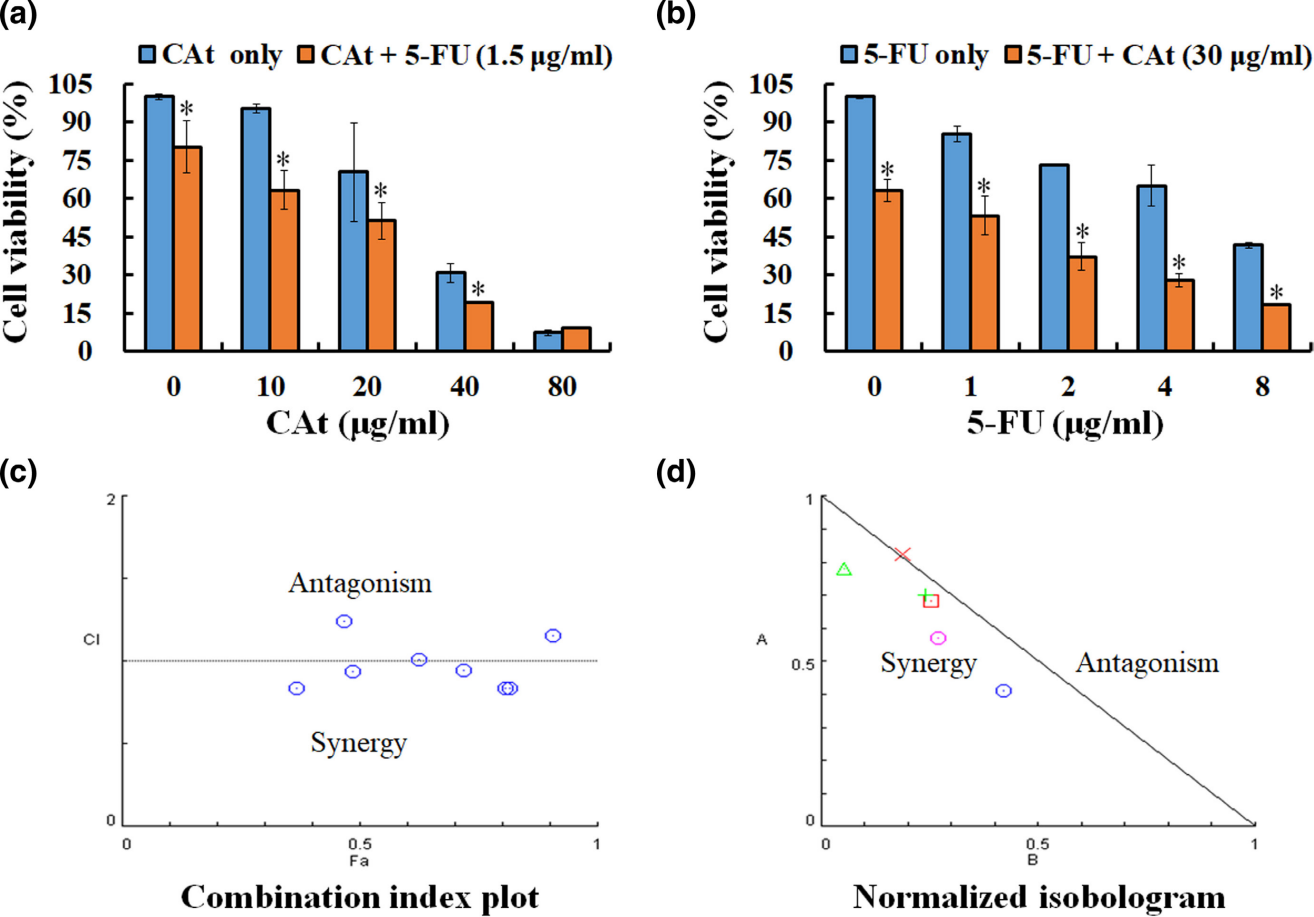
*Cedrus atlantica* extract (Cat) combined with 5‐fluorouracil (5‐FU) synergistically inhibited the growth of human colorectal adenocarcinoma (HT‐29) cells. HT‐29 cells were incubated with a combination of (a) CAt (0, 10, 20, 40, and 80 μg/ml) and/or 1.5 μg/ml 5‐FU; (b) 5‐FU (0, 1, 2, 4, and 8 μg/ml) and/or 30 μg/ml CAt for 48 h, and the cell viability measured using the 3‐(4,5‐dimethylthiazol‐2‐yl)‐2,5‐diphenyltetrazolium bromide (MTT) assay. The data are expressed as the mean ± SD. *p < .05 versus single drug group. Combination index (CI) plot (c) and normalized isobologram (d) were calculated and analyzed using CompuSyn software

The first‐line chemotherapy drug for CRC is 5‐FU; however, it has a short half‐life, which limits its effectiveness. Next, we evaluated whether combination treatment could enhance the growth inhibition of CRC cells. HT‐29 cells were treated with CAt (0–80 μg/ml) with or without 5‐FU or 5‐FU (0–8 μg/ml) with or without CAt, and cell viability was detected by the MTT assay. The results showed that the combination treatment of CAt and 5‐FU significantly decreased the viability of CRC cells compared with CAt or 5‐FU alone (Figure 2a,b). To investigate the drug interaction of CAt and 5‐FU, a combination index (CI) was calculated using Compusyn software, which quantitatively determined synergism (CI < 1), additive effect (CI = 1), and antagonism (CI > 1), respectively. As shown in Figure 2c,d, most of the combined drug doses showed a synergistic effect (CI > 1). Collectively, these data suggest that CAt in combination with 5‐FU synergistically inhibits the growth of CRC cells.

### 3.3 CAt arrested cell cycle at G0/G1 phase in HT‐29 cells

**FIGURE 3:**
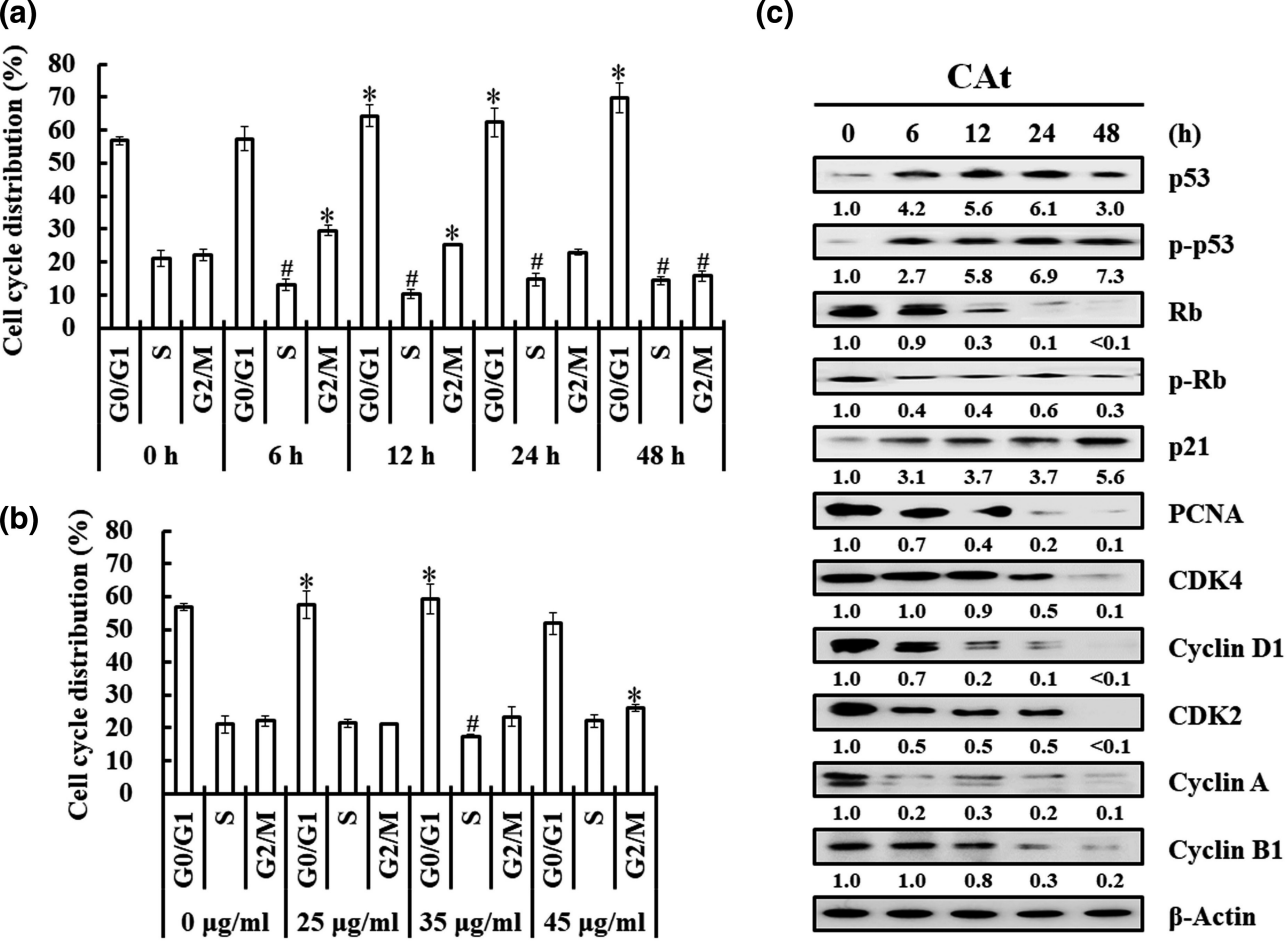
*Cedrus atlantica* extract (Cat) induced G0/G1 phase cell cycle arrest in human colorectal adenocarcinoma (HT‐29) cells. HT‐29 cells were treated with (a) 35 μg/ml CAt for 0, 6, 12, 24, and 48 h; (b) 0, 25, 35, and 45 μg/ml CAt for 24 h, and the cell cycle progression was determined by flow cytometry. The data are expressed as mean ± SD. *p < .05 versus control with significant increase. ^#^ *p* < .05 versus control with significant decrease. (c) After 35 μg/ml of CAt treatment for 0–48 h, the levels of proteins related to the cell cycle in HT‐29 cells were determined by western blot analysis

To determine whether the antiproliferation effect is attributable to the inhibitory effect of CAt on the cell cycle, cell cycle analysis was performed. HT‐29 cells were treated with IC_50_ concentration of CAt (35 μg/ml) for 0–48 h; CAt (0, 25, 35, and 45 μg/ml) for 24 h, stained with PI, and cell cycle distribution was analyzed by flow cytometry. The results showed that in comparison to 0 h, the cells treated with CAt for 48 h increased the percentage in G0/G1 phase from 56.77% to 69.82%, whereas the percentage of cells significantly decreased in S and G2/M phases (Figure 3a). Compared to treatment with various doses for 24 h, cell cycle arrest at the G0/G1 phase was induced by treatment with 25 and 35 μg/ml CAt (Figure 3b).

Furthermore, the expression level of cell cycle‐related proteins was determined by western blotting after the HT‐29 cells were treated with CAt (35 μg/ml) for 0–48 h. This indicated that the protein expression levels of p‐53/p‐p53 and p21 were increased, while the protein expression levels of Rb/p‐Rb and PCNA were decreased after CAt treatment (Figure 3c). In addition, the expression of cell cycle regulators, such as CKD4/cyclin D1, CKD2/cyclin A, and cyclin B1, was reduced in CAt‐treated cells in a time‐dependent manner. These findings suggest that CAt arrested the cell cycle at the G0/G1 phase in CRC cells by regulating the expression of p53/p21 and CDK4/cyclin D1 proteins.

### 3.4 CAt induced caspase‐dependent apoptotic cell death in HT‐29 cells

**FIGURE 4:**
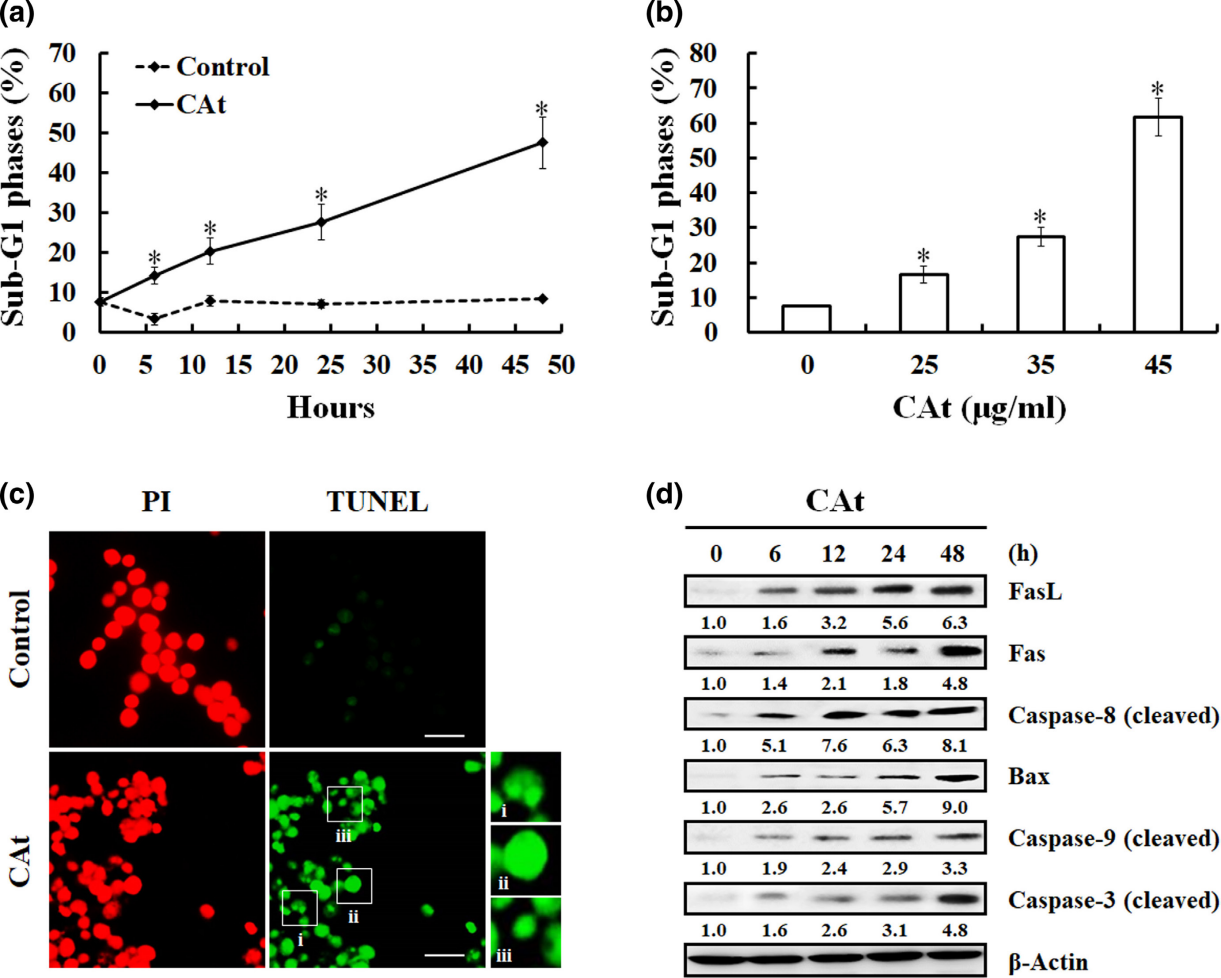
*Cedrus atlantica* extract (Cat) induced apoptosis cell death of human colorectal adenocarcinoma (HT‐29) cells via the activation of intrinsic and extrinsic pathways. (a, b) The CAt‐treated cells were collected and the percentage of cells at subG1 phase analyzed using flow cytometry. Data are presented as the mean ± SD. *p < .05 versus control. (c) After incubation with 35 μg/ml CAt for 48 h, the apoptotic cells were determined by the terminal deoxynucleotidyl transferase dUTP nick‐end labeling (TUNEL) assay and showed typical apoptotic morphology, such as chromatin condensation (i), DNA fragmentation (ii), and apoptotic body (iii). Scale bar = 20 µm. (d) The levels of apoptosis‐associated proteins were detected by the method of western blotting, and the blots were quantified using ImageJ software and compared with those of the control

HT‐29 cells were treated with CAt (35 μg/ml) for 0–48 h; CAt (0, 25, 35, and 45 μg/ml) for 24 h, and the percentage of cells in the subG1 phase was detected by flow cytometry. The results showed that in comparison with the control, the percentage of cells in the subG1 phase increased in a time‐ and dose‐dependent manner (Figure 4a,b). To determine whether cells underwent apoptosis, TUNEL staining was performed after HT‐29 cells were treated with 35 μg/ml CAt for 48 h. The TUNEL staining results showed that the percentage of cells with green fluorescence in the CAt‐treated groups was increased, and these cells formed apoptotic morphology, including chromatin condensation, DNA fragmentation, and apoptotic bodies (Figure 4c).

To further understand the specific mechanism of CAt leading to the apoptosis of HT‐29 cells, western blotting was used to detect the expression of apoptosis‐related proteins. Compared with the control, the protein levels of FasL/Fas/caspase‐8, Bax/caspase‐9, and cleaved caspase‐3 were increased (Figure 4d). These results showed that CAt effectively promoted apoptosis in CRC cells through extrinsic and intrinsic caspase‐dependent pathways.

### 3.5 CAt inhibited tumor growth of CRC in vivo

**FIGURE 5:**
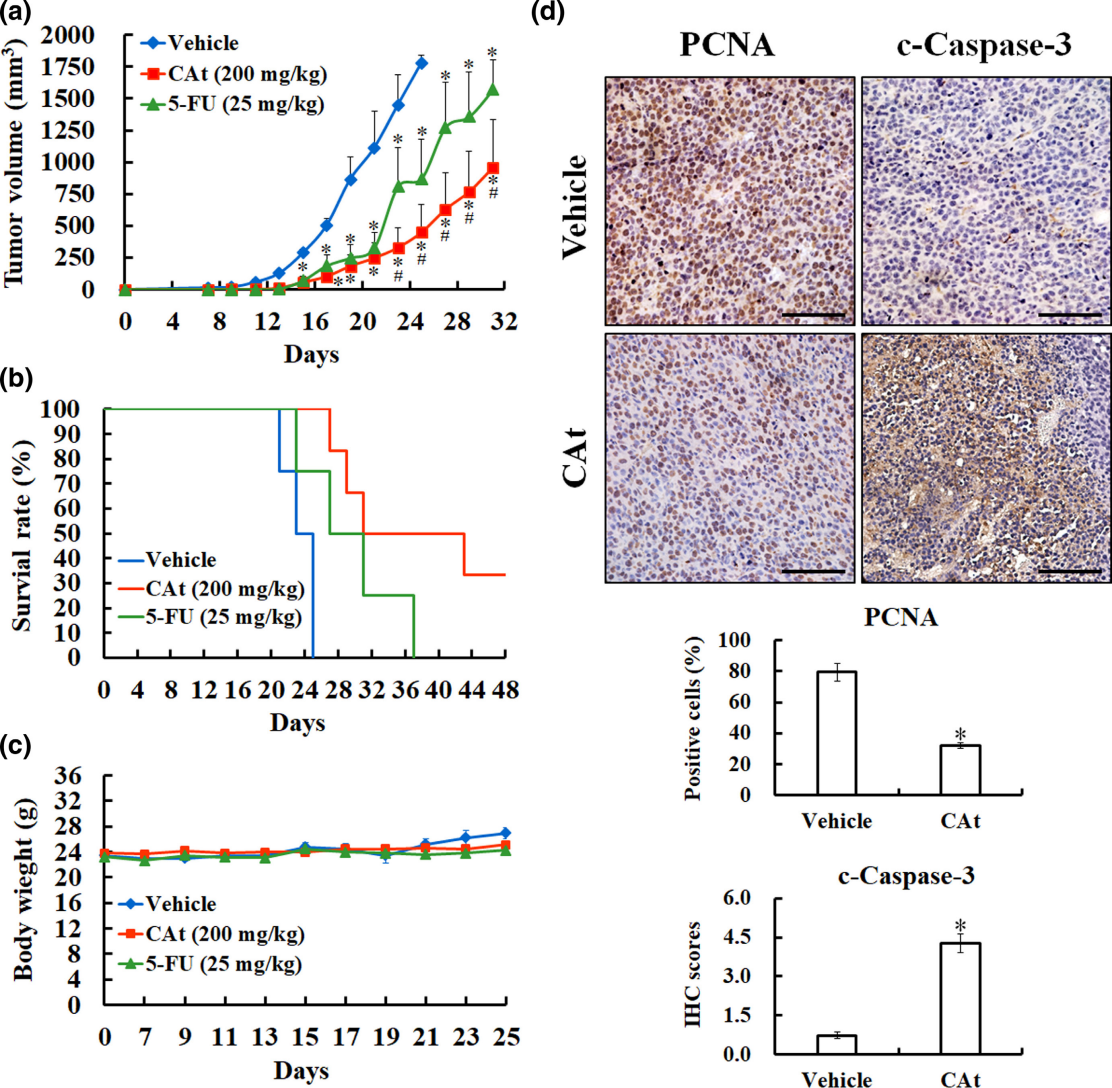
*Cedrus atlantica* extract (Cat)‐mediated inhibition of tumor growth in vivo. The tumor volume (a), survival rate (b), and body weight (c) of tumor‐bearing mice were recorded after administration of mineral oil (vehicle), CAt at 200 mg/kg, and 5‐fluorouracil (5‐FU) at 25 mg/kg. (d) The expression of proliferating cell nuclear antigen (PCNA) and cleaved caspase‐3 in tumor tissues was detected by immunohistochemical (IHC) assays (×400), quantified, and presented as percentages or IHC scores, respectively. Scale bar = 100 µm. All data are shown as the mean ± SEM. *p < .05 versus vehicle

A subcutaneous tumor model was established to determine whether CAt affected tumor growth in vivo, and the tumor volume and body weight were recorded in all mice. The tumor volume in the CAt‐treated group on day 25 was 449.54 ± 217.26 mm^3^, whereas that in the vehicle group was 1,780.64 ± 57.45 mm^3^ (Figure 5a). The survival time of tumor‐bearing mice was prolonged from 25 to 49 days after CAt treatment (*p* < .05; Figure 5b). This indicated that the tumor growth of implanted tumors was strongly suppressed after 38 days of CAt intervention (once every 2 days). In addition, the clinical drug 5‐FU had obvious inhibitory effects in the early stage of treatment, however, the tumor grew rapidly after the second treatment cycle. There was no significant difference in tumor volume between the CAt group and the 5‐FU group before day 21, but the CAt group showed more suppressive effects than the 5‐FU group after day 23. In this animal study, no obvious loss of body weight was observed in vehicle, CAt, or 5‐FU groups (Figure 5c).

Furthermore, the impact of CAt on cell proliferation and apoptosis in vivo was determined using IHC staining for PCNA and cleaved caspase‐3 (Figure 5d). The percentage of PCNA‐positive cells in CRC tumors was 79.3 ± 5.5% in the vehicle group and 32.0 ± 2.1% in the CAt group. On the other hand, in the vehicle group, the IHC score of cleaved caspase‐3 was 0.7 ± 0.1, whereas that in the CAt‐treated group was 4.3 ± 0.4. Overall, these findings suggest that CAt suppresses the growth of CRC tumors in vivo via the inhibition of proliferation and the induction of apoptosis, consistent with the in vitro results.

**FIGURE 6:**
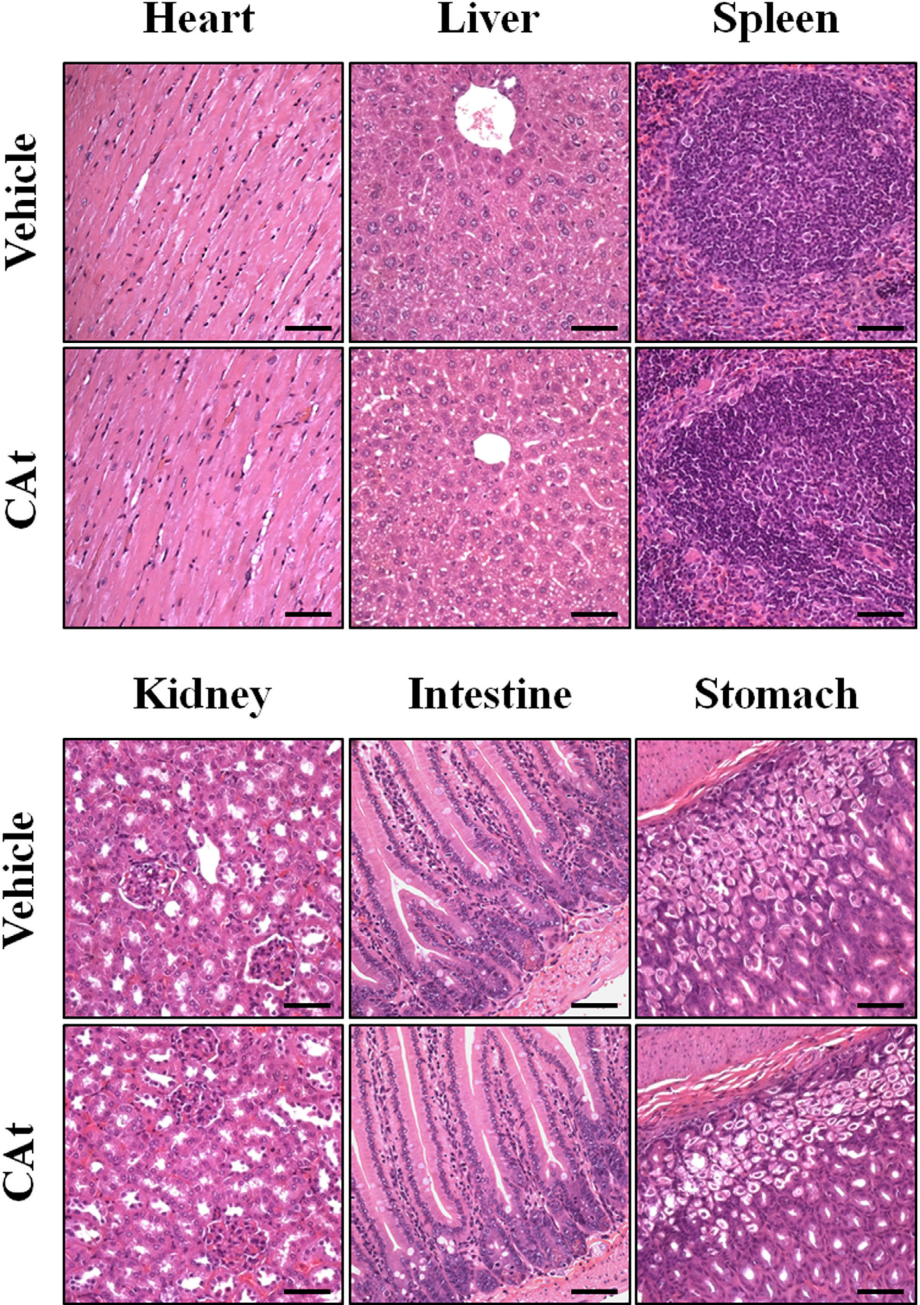
*Cedrus atlantica* extract (Cat) showed good tolerance with no significant toxicity after treatment. The mice were treated with 200 mg/kg CAt for 38 days once every 2 days, collected organs, including heart, liver, spleen, kidney, intestine, and stomach, and analyzed histological change using hematoxylin and eosin (H&E) staining. Scale bar = 50 µm

To evaluate the toxicity of CAt in vivo, we observed organ damage after a subcutaneous injection of 200 mg/kg CAt at 20 instances. The negative control animals received the same volume of solvent (mineral oil). No obvious morphological changes in organs in the heart, liver, spleen, kidney, intestine, and stomach were observed between the CAt and vehicle groups (Figure 6). The no‐observed‐adverse‐effect level for CAt in the 38‐day repeated injection study in mice was greater than 200 mg/kg body weight/2 days. These results suggest that the dose of CAt was well tolerated and effective for CRC treatment.

## 4 DISCUSSION

Herbal medicines have been used to treat cancer for millennia and are currently used alone or in combination with conventional therapies to treat various diseases (Sultana et al., 2014). It is known that plant‐based bioactive components exhibit anticancer activities in various ways, including changes in carcinogen metabolism, the activation of the immune system, the stimulation of DNA damage, the inhibition of cell cycle progression, and the induction of cell apoptosis. Compared with traditional anticancer agents, inducing cell growth arrest and apoptosis are the safest strategies for cancer treatment, as they are less toxic and have a lower risk of causing inflammation and side effects as a result of damaged and necrotic cells (Pfeffer & Singh, 2018; Samadi et al., 2015). In addition, natural products with potential anticancer activities are inexpensive compared to conventional anticancer agents (Seca & Pinto, 2018). Our previous studies indicated that a plant extract of *C. atlantica* (CAt) showed high potential for bioactivity against CRC screened from 24 plants through a drug screening platform based on the growth inhibition of CRC and normal cells. In the present study, we found that CAt inhibited the viability of CRC cells by suppressing cell proliferation and inducing apoptosis via extrinsic (death receptor) and intrinsic (mitochondrial) dependent apoptotic pathways. These results provide a foundation for the development of novel anti‐CRC drugs that are affordable and have little or no side effects.

The ability to replicate indefinitely and resist death is an important characteristic of cancer cells. Therefore, we first studied the effects of CAt on the proliferation and apoptosis of CRC cells. In the literature, the growth inhibition of *C. atlantica* is considered to be one of the mechanisms of its anticancer effects. For example, *C. atlantica* extracts have exhibited inhibitory activity against HepG2 and Mahlavu hepatocellular carcinoma (IC_50_ values 27.09 and 33.57 μg/ml, respectively) (Huang et al., 2020), DBTRG‐05MG and RG2 glioblastoma (IC_50_ value 46.59 and 47.96 μg/ml, respectively) (Chang et al., 2021), and K562 chronic myelogenous leukemia (IC_50_ value 59.76 μg/ml) (Saab et al., 2012) cell lines. In our study, we observed that CAt significantly decreased cell proliferation in HT‐29 and CT‐26 (IC_50_ values 31.21 and 19.77 μg/ml, respectively). Furthermore, CAt suppressed tumor growth and prolonged the life span of tumor‐bearing mice compared to the vehicle group. However, CAt showed lower cytotoxicity in normal cells in vitro and little or no toxicity in organs in vivo. Thus, these findings provide evidence regarding the use of CAt for the treatment of cancers and show potential for the development of anticancer drugs in the future.

Significant advances in CRC therapy have improved the overall survival rate of patients through the use of various promising drugs, such as oxaliplatin and 5‐FU, as well as antibodies, such as bevacizumab and cetuximab, inducing programmed cell death (Schwartz et al., 2004). In recent decades, 5‐FU has been used as the first‐line treatment for CRC (Vodenkova et al., 2020). However, the adverse effects and emergence of drug resistance remain a critical limitation to the clinical application of conventional chemotherapy (Hu et al., 2016). Recent progress has shown that combination treatment has many advantages over conventional treatment, including enhancing chemosensitivity and reducing the necessary drug dosage (Hu et al., 2016). Based on these results, in this study, we investigated the anticancer effect of the combination of CAt and 5‐FU. The data revealed that CAt improved the inhibitory effects of 5‐FU and showed that the combination of CAt and 5‐FU achieved better treatment effects than a single drug. Previous studies on glioblastoma showed that CAt enhanced the antiproliferative effects of temozolomide on DBTRG‐05MG and RG2 cells (Chang et al., 2021). Therefore, CAt may be a potentially effective antiproliferative drug or anticancer adjuvant agent combined with clinical drugs.

Several studies have shown that cell cycle progression controls cell proliferation (Dickson & Schwartz, 2009), and its dysfunction is a crucial stage in cancer development (Williams & Stoeber, 2012). Therefore, controlling cell cycle progression by inducing cell cycle arrest may be an appropriate strategy for cancer treatment (Carnero, 2002). In this study, flow cytometry analysis showed a significant accumulation of cells in the G0/G1 phase, along with a decrease in the percentage of cells in the S and G2/M phases after CAt treatment. The results implied that CAt induced cell cycle arrest at the G0/G1 phase in a time‐dependent manner, resulting in the discontinuing proliferation of damaged cells. It has been reported that p21, activated by the transcriptional factor p53, acts as an inhibitor of the CDK4/Cyclin D complex, which plays a vital role in the progression of the cell from the G1 phase to the S phase (Satyanarayana & Kaldis, 2009). Our data showed that CAt increased the protein expression levels of p53 and p21, and decreased the p‐Rb. In conjunction with these changes, the vital proteins CDK4/cyclin D1 in the G0/G1 phase were significantly inhibited. These results suggest that CAt may induce cell cycle arrest at the G0/G1 phase, resulting in the growth inhibition of CRC cells via the regulation of p53/p21 and CDK4/Cyclin D1.

Apoptosis (programmed cell death) plays a crucial role in the control of carcinogenesis and cancer treatment. Studies have indicated that treatment strategies, such as chemotherapy, radiotherapy, and surgery, usually involve inducing the apoptosis signaling pathway in most cancer cells (Ghobrial et al., 2005; Lee et al., 2014). The caspase protein family plays an important role in apoptosis and is produced as an inactive precursor (procaspase) in cells. Then, a series of caspases are activated, and cleaved caspases are produced after triggering the apoptotic pathway. Active initiator caspases, including caspase‐8 (extrinsic pathway) and caspase‐9 (intrinsic pathway), can activate other downstream caspases called executioner caspases (caspase‐3, −6, and −7). Caspase‐3 is the central enzyme responsible for plasma membrane reversion, nuclear and cytoplasmic protein degradation, and DNA fragmentation, which ultimately leads to cell death (Chinnaiyan, 1999; Degterev et al., 2003). In the present study, we found that CAt‐treated cells showed TUNEL‐positive results and apoptotic morphology, including chromatin condensation, DNA fragmentation, and apoptotic bodies. In addition, CAt induced apoptosis in HT‐29 cells through the activation of initiator and effector caspases (caspase‐8, −9, and −3). These findings indicate that CAt may induce HT‐29 cell apoptosis via extrinsic and intrinsic pathways.

In conclusion, the results of the present study showed that CAt inhibited the proliferation of HT‐29 cells by changing the cell cycle distribution, leading to cell cycle arrest at the G0/G1 phase via the regulation of p53/p21 and CDK4/cyclin D1. Moreover, CAt induced apoptosis through the activation of the extrinsic (FasL/Fas/caspase‐8) and intrinsic (Bax/caspase‐9) apoptotic pathways. In an animal study, subcutaneous treatment with CAt suppressed tumor growth in mice, which was well tolerated. In view of the above safe and effective anticancer effects, CAt may be exploited for the development of novel anticancer agents or dietary supplements against CRC.

## CONFLICT OF INTEREST

The authors declare no conflict of interest.

## ETHICAL APPROVAL

This study was approved by Institutional Animal Care and Use Committee (IACUC) of Chung Shan Medical University (Approval No. CSMU‐IACUC‐1543).

## Data Availability

The data that support the findings of this study are available from the corresponding author by reasonable request.
